# Prenatal famine exposure and estimated glomerular filtration rate across consecutive generations: association and epigenetic mediation in a population-based cohort study in Suihua China

**DOI:** 10.18632/aging.103397

**Published:** 2020-06-18

**Authors:** Wenbo Jiang, Tianshu Han, Wei Duan, Qiuying Dong, Wanying Hou, Huanyu Wu, Yue Wang, Zehui Jiang, Xinyi Pei, Yingying Chen, Ying Li, Changhao Sun

**Affiliations:** 1 Department of Nutrition and Food Hygiene, College of Public Health, Harbin Medical University, Harbin, Heilongjiang Province 150081, P. R. China; 2 The First Psychiatric Hospital of Harbin, Harbin, Heilongjiang Province 150081, P. R. China

**Keywords:** prenatal, famine exposure, transgenerational, DNA methylation

## Abstract

Prenatal malnutrition could promote renal dysfunction in adulthood, but it is unclear whether the detrimental effect could be transmitted to the next generation. We investigated whether famine exposure was associated with variation of estimated glomerular filtration rate(eGFR) in two generations and explored the mediation role of methylation alterations. The longitudinal analysis included 2909 participants from Suihua rural area. F1 and F2 generations were divided into non-famine and famine group based on their birth year and exposure status of their parents, respectively. The eGFR was calculated by using the chronic kidney disease epidemiology collaboration equation. We applied mixed-effect models to investigate the association between famine and ΔeGFR and tested blood DNA methylomes in 46 families across two generations. The mediation-analysis models were utilized to examine the mediation effect of methylation alterations on the famine-ΔeGFR association.

In mixed-effect models, famine exposure was associated with declined ΔeGFR level in F1(β:-8.32;95%CI:-11.51,-5.12) and in F2(β:-6.11;95%CI:-11.88, -0.43). Methylation850K BeadChip data showed only 19 of 961 F1 differentially methylated sites showed concordant alterations in F2. The mediation-analysis results showed methylation alterations on AGTR1 and PRKCA might mediate the famine-ΔeGFR association. Overall, prenatal famine exposure may have long-term effects on eGFR decline across consecutive generations which might be partly mediated by methylation alterations on AGTR1 and PRKCA.

## INTRODUCTION

Early-life malnutrition is a long-standing public concern in developing countries which may elevate the abnormal development of various organs [1–4] including kidney [5] in later life. A somewhat limited number of longitudinal studies have shown that nutritional deprivation of intrauterine or early postnatal environment had severely deteriorated effects on the kidney function in adulthood [6]. Besides, due to unavailable experimental data, the transgenerational effects on chronic kidney disease risk in adult offspring remain to be investigated.

As a natural experiment, famine provides a unique opportunity to explore the long-term association of prenatal malnutrition with renal function [2, 7]. The Great Chinese famine of 1959-1961 affected the whole nation and caused 30 million deaths and 30 million lost births during the period [8], and it was also superimposed on widespread chronic undernourishment [9]. Compared with other famines, Chinese famine persisted longer, affected more people, and swayed larger areas in China [10]. Thus, it offered a valuable opportunity to study the influence of the early-life famine on adult and offspring diseases [11].

An increasing amount of rodent studies have confirmed that exposure to famine in utero and postnatal life was associated with a high risk of T2D, obesity, metabolic syndrome, cognitive impairment, schizophrenia, etc. [12–15]. However, there was limited evidence indicating the association between famine exposure and chronic kidney disease (CKD). There were only a few epidemiological studies from the Chinese famine and Dutch famine, respectively, finding famine exposure during gestation and early postnatal period was in connection with a high concentration of proteinuria and microalbuminuria and was associated with the declined eGFR and greater risk of CKD in women [16]. Also, in animal models, we confirmed that prenatally undernourished rats might have a higher likelihood of compromising renal function [17], and maternally protein-energy malnutritional sheep has a greater risk of developing the renal disease in adult offspring [18]. However, very few evidence has directly investigated whether the potentially gestational effects on the declined eGFR level could be transmitted across generations.

Mounting evidence showed nutritional modifications might induce temporary or permanent epigenetic alterations that certainly have the potential to damage renal function dramatically. And several animal studies have supported the idea that environment-induced epigenetic changes (i.e. DNA methylation) could be inherited via the gametes in mammals. However, current evidence which indicated the environment could perennially influence the epigenome in humans is not extensive. In the present study, we aimed to analyze this longitudinal cohort in Northern China to provide direct evidence on the long-term impact of prenatal exposure to Chinese famine on eGFR alteration and investigate mediation effect of the specific DNA methylation loci on the association between famine and ΔeGFR in adulthood and offspring, to quantify indirect effects of maternal malnutrition during pregnancy.

## RESULTS

### Effect of prenatal famine exposure on kidney function in two cohorts

Table 1 summarized mean levels of study covariates at baseline and follow-up, respectively. At baseline, age, drink, and low working strength rate differed in F1 generation between two groups, whereas these covariates did not vary significantly in the F2 generation. And F1 and F2 generations in famine group had significantly higher FPG, 2-hPG, SBP, CRE and lower eGFR than those in the non-famine group, respectively. And the rates of eGFR<60 in the non-famine and famine groups were 12.76% and 23.21%, 14.70% and 16.74% in F1 and F2 generations, respectively. And then we applied the generalized linear mixed model to investigate the association between prenatal famine exposure and the risk of eGFR<60 in adulthood. After taking into account of the potential confounders, the odds ratios for eGFR<60 in two generations were exhibited in Table 2. In the F1 generation, the famine group had 1.80-fold (95% CI: 1.10, 3.36) higher risk compared with the non-famine group. In the F2 generation, however, parental exposure to famine was not shown to be associated with a higher rate of eGFR<60 which was probably due to the relatively young age in the F2 offspring at follow-up.

**Table 1:** Characteristics of the study variables at baseline and follow-up in Suihua cohort.

**Characteristic**	**F1 parent generation**			**F2 offspring generation**		
	**Non-famine (n=965)**	**Famine (n=916)**	***P*-Value**	**Non-famine (n=353)**	**Famine (n=675)**	***P*-Value**
Baseline						
Age (years)	52.7 (2.6)	53.1 (4.6)	<0.001	26.2 (5.2)	26.4 (4.5)	0.522
Men [n(%)]	671 (69.5)	569(62.1)	0.325	207 (58.6)	389 (57.6)	0.237
Energy(kcal/d)	2445 (1027)	2468 (1003)	0.512	2537 (845)	2501 (824)	0.431
Over high school level [n(%)]	146 (15.1)	129 (14.1)	0.203	42 (11.9)	74 (11.0)	0.523
Low working strength [n(%)]	279 (28.9)	233 (25.4)	0.032	98(27.8)	162 (24.0)	0.411
Smoker [n(%)]	341 (35.3)	321 (35.0)	0.851	128 (36.3)	226 (33.5)	0.151
Drinker [n(%)]	354 (36.7)	304 (33.2)	0.042	105 (29.7)	226 (33.5)	0.362
Hypertensive [n(%)]	459 (47.6)	495 (54.0)	0.012	76 (21.5)	171 (25.3)	0.091
Sport [n(%)]	279 (28.9)	244 (26.6)	0.326	85 (24.1)	176 (26.1)	0.332
BMI (kg/m^2^)	24.4 (3.1)	25.1 (3.2)	0.439	24.1 (4.2)	25.5 (3.2)	0.318
FPG (mmol/L)	4.5 (1.1)	4.8 (1.1)	0.021	4.3 (0.5)	4.5 (0.6)	0.024
2-hPG (mmol/L)	6.0 (1.8)	6.4 (2.6)	0.016	5.1 (1.2)	5.5 (1.3)	0.028
SBP (mmHg)	137.4 (23.3)	142.1 (21.2)	0.021	124.6 (16.0)	128.8 (14.1)	0.020
DBP (mmHg)	80.3 (13.8)	83.1 (16.1)	0.380	77.4 (11.0)	78.0 (10.6)	0.241
BUN (mmol/L)	5.4 (1.6)	5.8 (1.5)	0.022	4.9 (1.3)	5.1 (1.3)	0.029
UA (μmol/L)	284.4 (84.4)	294.9 (85.0)	0.022	280.1 (87.2)	284.3 (85.3)	0.853
CRE (mg/dL)	0.66 (0.14)	0.69 (0.18)	0.014	0.67 (0.14)	0.69 (0.15)	0.033
eGFR (ml/min/1.73 m^2^)	114.5 (30.3)	100.3 (24.5)	<0.001	130.2 (36.3)	115.0 (29.9)	0.007
Follow-up						
SBP (mmHg)	139.2 (21.1)	145.0 (22.3)	<0.001	126.9 (20.4)	132.5 (17.1)	0.014
DBP (mmHg)	82.2 (12.0)	86.0 (17.7)	0.011	77.6 (11.1)	80.2 (13.9)	0.332
BUN (mmol/L)	5.7 (1.4)	6.2 (1.5)	<0.01	5.1 (1.4)	5.4 (1.5)	0.017
UA (μmol/L)	288.4 (80.9)	300.4 (85.8)	<0.01	282.1 (82.5)	284.3 (85.9)	0.058
CRE (mg/dL)	0.67 (0.11)	0.71 (0.14)	<0.01	0.69 (0.17)	0.73 (0.15)	0.003
eGFR (ml/min/1.73 m^2^)	109.5 (32.8)	90.2 (28.6)	<0.001	118.2 (35.6)	100.9 (38.1)	0.001

Continuous variables are presented as the mean (SD). Generalized linear mixed models and χ^2^ test were used to probe for differences in continuous and dichotomous variables. eGFR, estimated glomerular filtration rate. BMI, Body mass index. FPG, Fast plasma glucose. 2-hPG, 2-hour postprandial glucose. SBP, Systolic blood pressure. DBP, Diastolic blood pressure. BUN, blood urea nitrogen. UA, Uric Acid. CRE, serum creatinine.

**Table 2:** The risk of eGFR<60 for parental and offspring in adulthood by famine exposure status.

	**F1 generation**		**F2 generation**	
	**Non-famine**	**Famine**	**Non-famine**	**Famine**
Rate, %	12.76	23.21	14.70	16.74
OR (95% CI)				
Model 1	1.00	1.66 (1.16, 2.77)	1.00	1.17 (0.83, 1.66)
Model 2	1.00	1.80 (1.10, 3.36)	1.00	1.26 (0.90, 1.75)

In the F1 generation, ORs (95% CIs) were calculated by the generalized mixed linear model with famine exposure (yes or no) as the fixed factor and family number as the random factor. Model 1 was adjusted for parental age and sex. Model 2 was adjusted as for Model 1 and for education, smoking, drinking, SBP, 2-hPG, working strength, daily energy intake, sport and BMI. In the F2 generation, ORs (95% CIs) were calculated by the generalized mixed linear model with parental famine exposure (yes or no) as the fixed factor and family number as the random factor. Model 1 was adjusted for offspring age and sex. Model 2 was adjusted as for Model 1 and for education, smoking, drinking, SBP, 2-hPG, working strength, daily energy intake, sport and BMI.

Moreover, Table 3 exhibited the results of linear mixed regression models which investigated the association of prenatal exposure to famine with ΔeGFR in two consecutive generations. The analyses results showed exposure to famine was associated with a β:-8.32 (-11.51, -5.12) lower ΔeGFR in the F1 generation and β:-6.11 (-11.88, -0.43) lower ΔeGFR in F2 generation after adjustment for age, sex, education, smoking, drinking, SBP, 2-hPG, working strength, daily energy intake, sport and BMI.

**Table 3:** Parental and offspring ΔeGFR in adulthood by famine exposure status.

	**F1 generation**		**F2 generation**	
	**Coefficient (95%)**	***P*-Value**	**Coefficient (95%)**	***P*-Value**
Model1	-9.95 (-12.77, -7.13)	<0.001	-6.15 (-11.83, -0.47)	0.035
Model2	-8.32 (-11.51, -5.12)	<0.001	-6.11 (-11.88, -0.43)	0.034

In the F1 generation, coefficients and their 95% CIs were calculated by the mixed linear model with famine exposure (yes or no) as the fixed factor and family number as the random factor. Model 1 was adjusted for parental age and sex. Model 2 was adjusted as for Model 1 and for education, smoking, drinking, SBP, 2-hPG, working strength, daily energy intake, sport and BMI. In the F2 generation, coefficients and their 95% CIs were calculated by the mixed linear model with parental famine exposure (yes or no) as the fixed factor and family number as the random factor. Model 1 was adjusted for offspring age and sex. Model 2 was adjusted as for Model 1 and for education, smoking, drinking, SBP, 2-hPG, working strength, daily energy intake, sport and BMI.

### Effect of prenatal famine exposure on nutrition-sensitive DMSs in two consecutive generations

**Figure 1:**
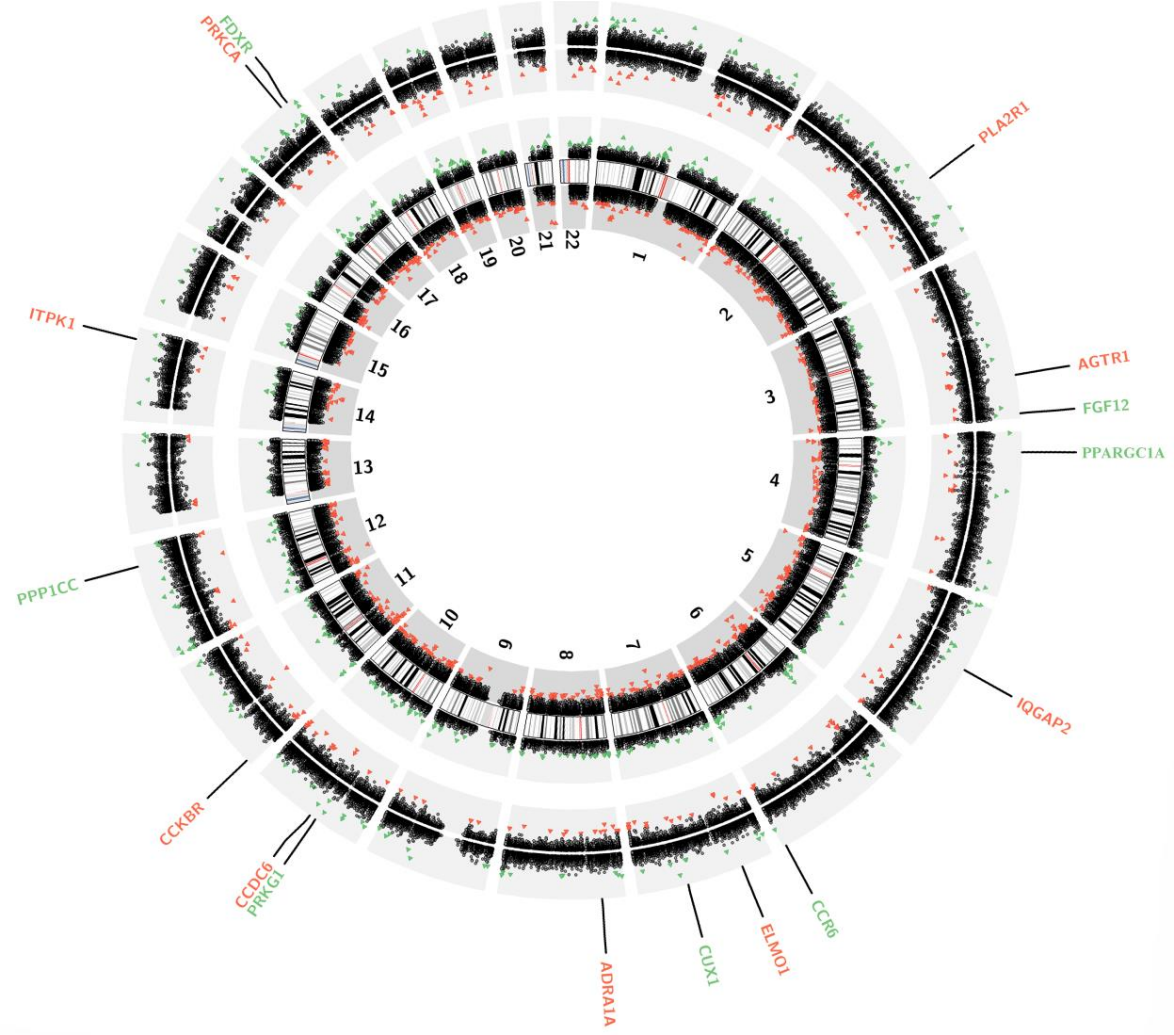
**Genome-wide DNA methylation changes in the participants prenatally exposed to famine.** Circos plot depicting the entire autosomal complement. Each dot marks the location of the Illumina Methylation EPIC Bead probe along the genome. The dots on the innermost black ring and the next outermost black circle represent the CPG sites in the F1 generation and F2 generation, respectively. Green and red dots represent significantly hypo-methylated and hyper-methylated differentially methylated sites. The innermost black ring with vertical lines represents autosome ideograms annotated with the chromosomal number.

In the F1 generation, 961 differentially methylated sites (DMSs) (|Δβ|>0.05 and adjusted *P*<0.05) including 601 hypermethylated and 360 hypomethylated DMSs were identified. In the F2 generation, 503 DMSs (|Δβ|>0.05 and adjusted *P*<0.05) including 146 hypermethylated and 357 hypomethylated DMSs were identified. We constructed a genome-wide map of all autosomal DMSs (Figure 1). And only 19 of 961 F1 DMSs showed concordant alterations in F2 cohort, which may suggest that these DNA methylation alterations after prenatal famine exposure might be subject to intergenerational transmission. According to the annotation, 17 DMSs were physically located within 16 unique genes including genes with a critical role in kidney development (CUX1, PPARGC1A) as well as glomerular protection (ELMO1) and renal cell survival (AGTR1) (Supplementary Table 4).

### Nutrition-sensitive DMSs correlated with ΔeGFR in two consecutive generations

**Figure 2:**
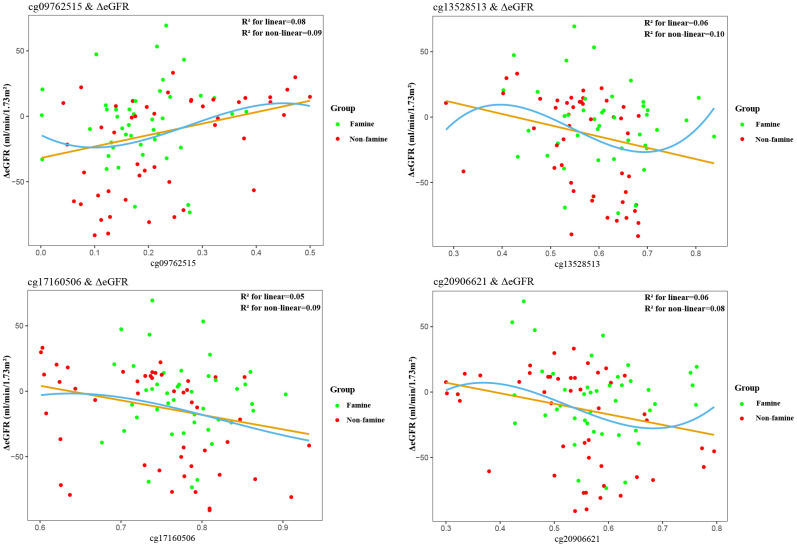
**Association between epigenetic dysregulation in parental blood and ΔeGFR after fetal malnutrition.** Correlation graphs of (y axis) ΔeGFR and (x axis) β values showing association between DNA methylation on cg09762515 (**A**), cg13528513 (**B**), cg17160506 (**C**), cg20906621 (**D**)and ΔeGFR in F1 generation.

**Figure 3:**
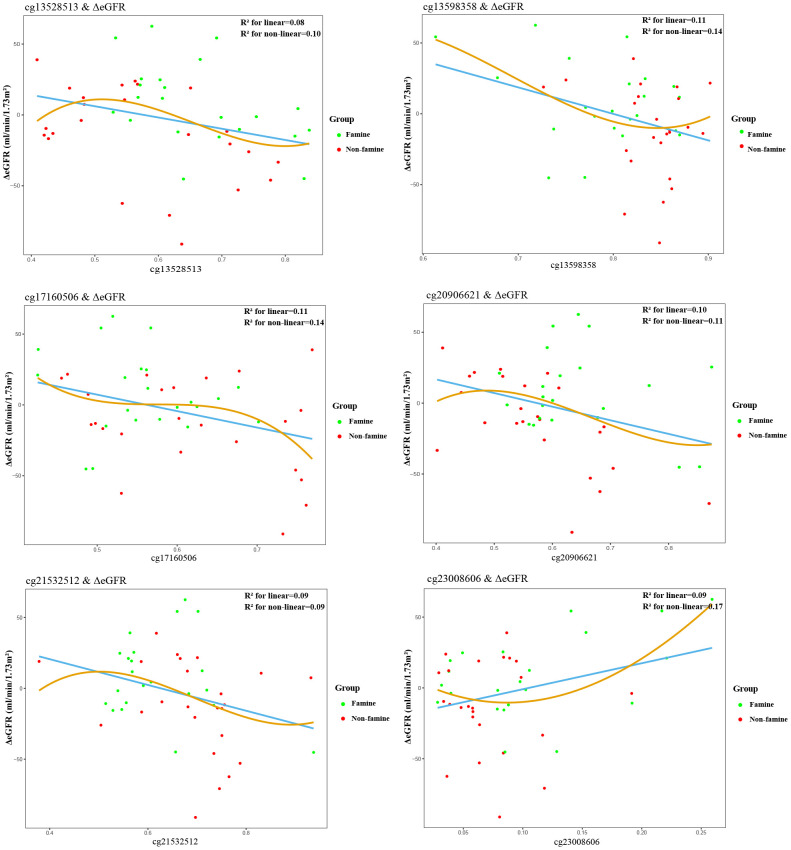
**Association between epigenetic dysregulation in offspring blood and ΔeGFR after fetal malnutrition.** Correlation graphs of (y axis) ΔeGFR and (x axis) β values showing association between DNA methylation on cg13528513 (**A**), cg13598358 (**B**), cg17160506 (**C**), cg20906621 (**D**), cg21532512 (**E**), cg23008606 (**F**) and ΔeGFR in F2 generation.

Partial correlations revealed that among 19 methylated CpGs, 4 DMSs (cg09762515, cg13528513, cg17160506, cg20906621) were significantly correlated with ΔeGFR in F1 generation (Figure 2), 6 DMSs (cg13528513, cg13598358, cg17160506, cg20906621, cg21532512, cg23008606) were significantly relevant to ΔeGFR in F2 generation (Figure 3). Adjustment for key demographic factors such as age and sex overall resulted only in minor changes in the association between DMSs and ΔeGFR.

### Key nutrition-sensitive DMSs were validated their differences in additional samples

Two DNA fragments from AGTR1 and one DNA fragment from PRKCA were analyzed, and the site-specific methylation level within each DNA fragment was quantified by targeted bisulfite sequencing to validate differential DNA methylation in an additional sample set. PCR primers were designed using the Methylation Primer software based on the bisulfite converted DNA. As shown in Supplementary Table 5, three DMSs were validated in 390 F1 participants and 210 F2 participants.

### Mediation effects of methylation sites on the famine-ΔeGFR association

Mediation-analysis step 1 was conducted to investigate the mediator role of these three CpG sites in F1 generation in the correlation between F1 famine exposure and F1 ΔeGFR. As shown in Table 4, the total effect showed prenatal exposure to famine was associated with lower ΔeGFR in F1 generation after adjustment for parental characteristics. The mediation effects of methylation alteration at cg13528513, cg20906621 and cg17160506 were 23.20, 26.52 and 21.28 percent (*P*=0.031, *P*=0.016, *P*=0.009) of the total effect, respectively.

**Table 4:** Associations between prenatal famine exposure and ΔeGFR in adulthood with the mediation of DNA methylation in the F1 generation.

**Predictor(F1)**	**Mediator(F1)**	**Outcome(F1)**	**Total effect Estimate,(95% CI)**	**Proportion via mediation Estimate,%**	**Sensitivity analysis**	
					R^2*	**R^2~**
Famine exposure	cg13528513	ΔeGFR	-0.26 (-0.46 to -0.06)	23.20	0.16	0.112
Famine exposure	cg20906621	ΔeGFR	-0.30 (-0.52 to -0.08)	26.52	0.16	0.112
Famine exposure	cg17160506	ΔeGFR	-0.22 (-0.41 to -0.02)	21.80	0.09	0.055

The predictor is F1 prenatal exposure to famine, the mediator is F1 DNA methylation, and the outcome is F1 ΔeGFR. R^2*, the proportion of residual variances. R^2~, the proportion of original variances that were explained by the omitted confounding

Mediation-analysis step 2 was performed to demonstrate the famine associated methylation variations could be transgenerational. As shown in Table 5, parental prenatal exposure to famine was associated with 1.6-2.0, 1.3-3.1 and 1.8-2.3 percent decrease in the methylation level at cg13528513, cg20906621 and cg17160506 in the F2 generation. Of the total effect, 28.66-29.31, 25.38-32.65 and 29.97-30.98 percent were mediated by methylation alterations at cg13528513, cg20906621 and cg17160506, respectively.

**Table 5:** Associations between F1 prenatal famine exposure and F2 DNA methylation with the mediation of F1 DNA methylation.

**Predictor(F1)**	**Mediator(F1)**	**Outcome(F2)**	**Total effect Estimate,(95% CI)**	**Proportion via mediation Estimate,%**	**Sensitivity analysis**	
					R^2*	**R^2~**
Paternal exposure	cg13528513	cg13528513	-0.016 (-0.030 to -0.003)	28.66	0.16	0.112
Paternal exposure	cg20906621	cg20906621	-0.013 (-0.025 to -0.001)	25.38	0.04	0.033
Paternal exposure	cg17160506	cg17160506	-0.018 (-0.028 to -0.008)	30.98	0.09	0.067
Maternal exposure	cg13528513	cg13528513	-0.020 (-0.032 to -0.009)	29.31	0.09	0.066
Maternal exposure	cg20906621	cg20906621	-0.031 (-0.048 to -0.014)	32.65	0.16	0.112
Maternal exposure	cg17160506	cg17160506	-0.023 (-0.030 to -0.015)	29.97	0.09	0.067

The predictor is F1 prenatal exposure to famine, the mediator is F1 DNA methylation, and the outcome is F1 ΔeGFR. R^2*, the proportion of residual variances. R^2~, the proportion of original variances that were explained by the omitted confounding

Mediation-analysis step 3 was implemented to the mediator role of these three CpG sites in F2 generation in the relationship between F1 famine exposure and F2 ΔeGFR. As shown in Table 6, our study uncovered that the association between parental prenatal exposure to famine and ΔeGFR in the offspring generation was significant. And the mediation effects of cg13528513, cg20906621 and cg17160506 on the ΔeGFR were significant (*P*=0.034, *P*=0.047, *P*=0.043).

**Table 6:** Associations between F1 parental prenatal famine exposure and F2 ΔeGFR in adulthood with the mediation of F2 DNA methylation.

**Predictor(F1)**	**Mediator(F2)**	**Outcome(F2)**	**Total effect Estimate, (95% CI)**	**Proportion via mediation Estimate,%**	**Sensitivity analysis**	
					R^2*	**R^2~**
Paternal exposure	cg13528513	ΔeGFR	-0.05 (-0.09 to -0.02)	8.77	0.16	0.112
Paternal exposure	cg20906621	ΔeGFR	-0.13 (-0.23 to -0.03)	11.47	0.16	0.112
Paternal exposure	cg17160506	ΔeGFR	-0.10 (-0.17 to -0.03)	8.60	0.09	0.066
Maternal exposure	cg13528513	ΔeGFR	-0.11 (-0.17 to -0.05)	8.13	0.09	0.067
Maternal exposure	cg20906621	ΔeGFR	-0.09 (-0.16 to -0.01)	10.10	0.09	0.067
Maternal exposure	cg17160506	ΔeGFR	-0.06 (-0.09 to -0.03)	9.10	0.09	0.066

The predictor is F1 prenatal exposure to famine, the mediator is F1 DNA methylation, and the outcome is F1 ΔeGFR. R^2*, the proportion of residual variances. R^2~, the proportion of original variances that were explained by the omitted confounding

The mediation-analysis in our study relied on the following negligible assumption of point identification. Since it is impossible to exclude the existence of missing variables in the relationship between confounding mediators and results, many studies do not satisfy the sequential ignorability hypothesis. Therefore, sensitivity analysis is recommended to determine to what extent the presence of confounders may affect the estimates. In our study, the results of sensitivity analysis showed that the proportion of residual variance (R^2*) and the proportion of original variance (R^2~) explained by ellipsis confusion were 0.040 ~ 0.160 and 0.033 ~ 0.112, respectively.

## DISCUSSION

In this large longitudinal cohort study with 2909 participants across consecutive generations in Northern China, we novelly found that prenatal exposure to Chinese famine significantly decreased eGFR in adulthood in two consecutive generations. Besides, based on the mediation-analysis model, the famine-ΔeGFR association appeared to be mediated considerably by methylation alterations in AGTR1 and PRKCA.

The findings of our present study are consistent with previous reports in the Chinese famine studies on undernutrition in early-life and renal function [7]. Two intriguing studies based on Chinese famine indicated famine exposure was linked to an enhanced risk (OR:1.54; 95% CI: 1.04, 2.28) of higher proteinuria level among women exposed to famine. And prenatal exposure to famine was associated with declined eGFR and a higher risk of CKD in women [7, 16]. Moreover, in an animal model, we found that prenatally undernourished rats might have a higher risk of compromising kidney function [17]. Also, our present study provided direct evidence that prenatal exposure to famine was associated with declined eGFR, which could be supported by the studies above.

To the date, there has been limited research on the potential transgenerational effects of prenatal exposure to malnutrition on renal-related outcomes. As this study investigated the transgenerational programming of nephron deficits, we will focus on the transmission of cardiovascular disease and metabolic disease in models that are known to program parental nephron deficits. We [19] and others [20–22] have reported the transmission of metabolic diseases such as T2D and abdominal obesity across generations previously. And to our knowledge, human evidence about whether early-life famine could transgenerationally affect kidney function in the next generation has not yet been reported. This large population-based cohort is the first direct evidence supporting the viewpoint that prenatal exposure to famine played an influential role in decreased eGFR in adulthood, and this detrimental effect could be transmitted to the next generation.

Whereas potential biological mechanisms for this multigenerational effect of prenatal exposure to famine on the declined eGFR are still not elucidated, but entertained interpretation such as the role of epigenetics may be involved. A few recent reports suggested that markers of vascular/endothelial dysfunction were related to early-life nutritional exposure through epigenetic modifications in the kidney, such as DNA methylation alterations [23] or histone structure modifications [24, 25]. And a pregnancy exposure to famine in a woman carrying a female fetus could straightly affect her grandchildren while germline in males could also be influenced. Indeed, the evidence from Dutch famine study supported that DNA methylation acted as a mediator of the association between prenatal adversity and risk factors for metabolic disease in adulthood [26]. Nevertheless, until now, we are still unable to find out whether persistent epigenetic differences occur in the next generation, thus result in the declined renal function in F2 adulthood. Whereas our results are broadly supported by findings from several animal studies, which have found transgenerational effects of maternal undernutrition in F1 could damage cardiovascular and renal function in the F2 generations via the altered epigenetic modifications through germlines of parents [27, 28]. Therefore, in the aspect of biological mechanism, we inferred that the observed transgenerational association between F1 prenatal exposure to famine and F2 impaired renal function might have been ascribed to the transgenerational alteration of epigenome pattern through the germline cell of the F1 generation.

Therefore, we tested blood DNA methylomes in 138 subjects across two generations. And the result showed that only 19 of 961 F1 DMSs showed concordant alterations in the F2 generation, which may indicate that these nutrition-sensitive DNA methylation alterations could be subject to intergenerational transmission. And only three of 19 methylation sites were correlated with ΔeGFR, locating at AGTR1 and PRKCA which have been proved to be associated with a renal injury accompanied with renal dysfunction [29–31]. Our study performed three steps mediation-analysis to test the viewpoint that methylation alterations in the three CpG sites were mediators of the F1 prenatal exposure to famine on F2 ΔeGFR. First, step 1 result showed that prenatal exposure to famine was significantly associated with ΔeGFR in the F1 generation, which could be mediated by methylation changes at the three CpG sites. Second, step 2 result indicated that the methylation alterations in F1 generation could partially be transmitted to their next generation. Third, step 3 result demonstrated that the relationship between F1 prenatal exposure to famine and F2 ΔeGFR could be partly ascribed to the methylation alterations at AGTR1 and PRKCA, which seemed that these three epigenetic marks might escape reprogramming during the period of the embryonic development.

Notable methodological strengths of our study were exhibited as follows. First of all, the present report firstly discussed the mediating effect of methylation alterations on the association between famine and eGFR alteration. Second, this longitudinal cohort was the first large population-based study to investigate the transgenerational effects of parental prenatal famine on eGFR alteration of next-generation in adulthood. Yet this present study also has several certain limitations to be taken into account. First, in contrast to other famines, Chinese famine persisted for a more extended period (about three years) [32] which signified that the famine subjects underwent the intrauterine and infancy stage. But the previous report indicated that the intrauterine stage was regarded as the dominating critical period [33]. Second, Children who were born with deficient birth weight are at risk for the development of chronic kidney disease [34]. And there is a dose-response relationship between worsening in eGFR and birth weight/gestational age [35]. It is deeded a limitation for lacking the data of birthweight and gestational age in the F2 generation. Hence, it is significant to conduct a follow-up study in the future for more comprehensive data. The last limitation is that the CpGs for the mediation-analysis were found only in our Suihua cohort, but some of these differentially methylated sites have been reported in previous famine studies using the Illumina 450k arrays [36–38], such as those located in solute carrier family 38 member 2 (SLC38A2), phospholipid phosphatase 2 (PPAP2C), zinc finger protein 385A (ZNF385A) and family with sequence similarity 150 member B (FAM150B). These consistent observations further demonstrated the impact of famine on the epigenetic changes. Besides, in future research, we would include more cohorts for maximumly eliminating possibilities of overinterpretation and perform animal studies to validate the intergenerational transmission of these DNA methylation sites.

Overall, we discovered that prenatal exposure to famine was associated with declined eGFR across two consecutive generations of Chinese adults. The significant mediation effects of methylation alterations on the association between famine exposure and eGFR in both generations were found which could suggest that prenatal-time window requires the highest priority in obtaining nutritional relief and also support further targeted research along this line.

## MATERIALS AND METHODS

### Participants and selection

The participants were recruited from the Suihua rural region of Heilongjiang Province, which is located in the northeast of China and suffered from severe famine from 1959 to 1961. At baseline, the participants were selected based on the household unit, including two generations (i.e. F1 parents and F2 offspring) in 2012. F1 parents born between Oct 1st 1959 and Sep 30th 1961 were classified as prenatal famine exposure group, and parents born between Oct 1st 1956 and Sep 30th 1958 and born between Oct 1st 1962 and Sep 30th 1964 were classified as the non-famine group. Correspondingly, F2 offspring were classified as famine and non-famine groups according to their parents' exposure status. And a total of 1173 households with F1 born between Oct 1st 1956 and Sep 30th 1964 were selected in the study at baseline. In 2017, the first follow-up survey was completed with a follow-up rate of 89.1%. 2909 participants, including 1881 F1 parent and 1028 F2 offspring with measurement of serum biochemical indexes were enrolled in this study. The Ethical Committee of Harbin Medical University approved the study protocol and written informed consent was obtained from all the participants. And this trial was registered at the Chinese Clinical Trial Registry as ChiCTR-ECH-13003644.

### Questionnaire and anthropometric measurement

At baseline and follow-up, a standardized face-to-face interview by trained staff covered socio-demographic characteristics (e.g. age, sex, education level) and lifestyles (e.g. tobacco smoking, drinking, physical activity) and a food-frequency questionnaire (FFQ) which was used to collect dietary information on intake frequency and the average consumption of each food item in usual. And the availability and reproducibility of the FFQ were assessed in our previous study [19]. The daily energy intake of every participant was estimated using the Food Nutrition Calculator (V1.60; Chinese Center for Disease Control).

The physical examination was conducted at the same time as the questionnaire. Systolic and diastolic blood pressure were obtained with the participants in the sitting position. Two consecutive blood pressure measurements were conducted after five minutes of rest. We also measured participants' height without footwear and weight in light clothing and calculated the body mass index as weight divided by the square of height. Fasting blood samples and postprandial blood samples were collected using for laboratory measurements. Fast plasma glucose (FPG), 2-hour postprandial glucose (2-hPG), blood urea nitrogen (BUN), uric acid (UA), and serum creatinine (CRE) were determined using an automatic biochemical analyzer (Hitachi 7100; Hitachi Ltd). Hypertension was defined as SBP/DBP≥140/90 mmHg or current therapy for definite diagnosis of hypertension. T2D was defined as FPG≥7.0 mmol/L or 2-hPG≥11.1 mmol/L or current therapy for definite diagnosis of T2D. The eGFR values were calculated from CRE, age and sex by using the chronic kidney disease epidemiology collaboration equation (CKD-EPI equation):141×(CRE/0.9)^-0.411^×(0.993) ^age^ (Male, CRE≤0.9); 141×(CRE/0.9)^-1.209^×(0.993) ^age^ (Male, CRE>0.9); 144×(CRE/0.7)^-0.329^×(0.993) ^age^ (Female, CRE≤0.7); 144×(CRE/0.7)-^1.209^×(0.993) ^age^ (Female, CRE>0.7). And ΔeGFR was defined as eGFR at baseline - eGFR at follow-up.

### Association between famine exposure and ΔeGFR

Continuous data were expressed as the mean ± standard deviation, and categorical data were presented as proportion. Association between prenatal exposure to famine and ΔeGFR, in both generations, was examined by using a linear mixed model. In this model, the family number was the random factor and famine exposure (yes or no) was the fixed factor. We adjusted for the following confounders: age (continuous), sex (male or female), education level (≤high school or > high school), energy intake (continuous), smoking (never, past, current), drinking (yes or no), working strength (low, medium, high), SBP (continuous), 2-hPG (continuous) and BMI (continuous).

### Genome-wide DNA methylation profiling

Blood samples collected from 46 families in 2012 (92 adult F1 participants (n=46 famine; n=46 non-famine) and 46 F2 offspring (n=23 famine; n=23 non-famine)) were measured genome-wide DNA methylation profiling using the Illumina Infinium HumanMethylation850K BeadChip (Illumina Inc, USA). The basic characteristics of these subjects are listed in Supplementary Table 1. First, DNA was isolated from whole blood leukocytes using DNeasy Blood and Tissue Kit (Qiagen) and was stored at -80 °C for further research. Approximately, 500 ng of genomic DNA from each sample was utilized for sodium bisulfite conversion with EZ DNA methylation Gold Kit (Zymo Research, USA) leaving methylated cytosine unchanged and converting unmethylated cytosine to uracil. Then DNA samples were distributed uniformly in different arrays according to famine exposure status, generation and sex, to minimize batch (microarray chip) effects.

### Data processing and statistical analysis of Illumina 850K methylation data

Raw genome-wide methylation data were processed using the R package minfi and assessed for basic quality control metrics. In each sample, probes with insignificant detection p-value above background control probes (detection p-value > 0.01) were removed. And probes with an SNP in the single base extension site were excluded. Also, we removed heterosomal probes from the raw methylation values and retained unique sequences for 755,464 autosomal probes which were available for analysis. A total of 138 samples passed quality control metrics and were further considered for statistical analyses. The HumanMethylation850k BeadChip contained two assay types (type I and type II) which utilized different probe designs. To correct distinct profiles produced by these two assay types, we performed a beta mixture quantile normalization method (BMIQ) on the normalized data to adjust the β-values of type II design probes into a statistical distribution characteristic of type I probes. Methylation β-value (ranging from 1 to 0, corresponding to fully methylated and completely unmethylated sites, respectively) represented the ratio of the signals of the methylated allele to the sum of the signals of the methylated and unmethylated alleles.

Our report implemented the comparison of famine and non-famine groups in two steps: (I) famine versus non-famine in F1 and (II) famine versus non-famine in F2. We performed a multivariable regression model to differentiate the role of prenatal malnutrition on methylation from the potential confounders of sex, smoking and BMI at these methylation sites. Once prenatal malnutrition in the F1 generation or parental prenatal malnutrition in F2 generation played a significant role in DNA methylation variation, p-values would be significant. Then the p-values would be done correction of FDR with the Benjamini-Hochberg method (adjusted p-value =p-value × Max rank/ rank of p-value). And the adjusted p-value <0.05 was considered statistically significant.

### Targeted bisulfite sequencing analysis of methylation level for target CpG sites

The whole blood samples of 195 families (390 adult F1 participants (n=194 famine; n=196 non-famine) and 210 F2 offspring (n=83 famine; n=127 non-famine)) underwent targeted bisulfite sequencing analysis for target CpG sites. The basic characteristics of these subjects are listed in Supplementary Table 2. After PCR amplification of target CpG sites and library construction, the products were sequenced on Illumina MiSeq Benchtop Sequencer (CA, USA). All samples achieved a mean coverage of > 1000X. Tested CpG sites were named as its relative distance to the transcriptional start site (TSS). The methylation degree of each CpG site was calculated as the percentage of methylated cytosine relative to the total cytosine. PCR was performed to amplify the three targeted DNA sequences and the relative distance from the TSS, amplification primers, and product size of these CpG sites could be found in Supplementary Table 3. For targeted bisulfite sequencing assays data, the independent variables such as sex, smoking, BMI, incorporating prenatal malnutrition status would be included in the multivariable regression model. Likewise, adjusted p-value <0.05 was considered statistically significant.

### Methylation/ΔeGFR correlations in F1 and F2 generation

Mediation models were performed to evaluate the role of DNA methylation as potential mediators of the association between famine and ΔeGFR in F1 and F2 generation. Famine or parental famine-exposure status as the predictor (X), CpG sites the mediators (M), ΔeGFR the outcome (Y). Then three steps mediation-analysis was performed to demonstrate that these methylation levels of CpG sites were the mediator of F1 famine exposure on F1 ΔeGFR (step 1), transgenerational (step 2), and the mediator of F1 famine exposure on F2 ΔeGFR (step 3). And mediation-analysis were performed using R package lavaan to obtain the total effects and mediation effects. Sensitivity analysis was completed within the function, and mediating effects were estimated to explore the robustness of the results in the case of violation of the following negligible assumption. The sensitivity parameters were residual variance proportion (R^2*) and original variance proportion (R^2~).

All statistical tests were performed using the R 3.4.4 software (The R Foundation for Statistical Computing) and SPSS Statistics v23.0 (IBM Corp. USA) and p-value < 0.05 was considered statistically significant.

## Supplementary Material

Supplementary Table
