# Supplementary material for: Prenatal famine exposure and estimated glomerular filtration rate across consecutive generations: association and epigenetic mediation in a population-based cohort study in Suihua China
Source: Aging (Albany NY). 2020 Jun 18;12(12):12206–21. doi: 10.18632/aging.103397 (PMC7343514; doi:10.18632/aging.103397)
Supplement: Supplementary Table [file aging-12-103397-s001..pdf]

## SUPPLEMENTARY TABLES

**Supplementary Table 1. Basic characteristics of the studied subjects for 850k Methylation BeadChip.**

| Characteristics | Studied subjects for 850k Methylation BeadChip | | | | | |
|---|---|---|---|---|---|---|
| | F1 parent generation | | | F2 offspring generation | | |
| | Non-famine (n=46) | Famine (n=46) | *P* | Non-famine (n=23) | Famine (n=23) | *P* |
| Age (years) | 52.7 (4.6) | 52.2 (2.5) | 0.014 | 26.0 (4.6) | 25.1 (4.2) | 0.664 |
| Men [n(%)] | 22 (47.8) | 22 (47.8) | 0.552 | 13 (56.5) | 14 (60.9) | 0.222 |
| Energy (kcal/d) | 2367 (913) | 2418 (966) | 0.622 | 2524 (863) | 2638 (883) | 0.325 |
| Over high school level [n(%)] | 18 (39.1) | 21 (45.7) | 0.667 | 9 (39.1) | 8 (34.8) | 0.585 |
| Low working strength [n(%)] | 13 (28.3) | 11 (23.9) | 0.145 | 7 (30.4) | 7 (30.4) | 0.561 |
| Smoker [n(%)] | 16 (34.8) | 20 (43.5) | 0.322 | 10 (43.5) | 11 (47.8) | 0.332 |
| Drinker [n(%)] | 18 (39.1) | 20 (43.5) | 0.221 | 13 (56.5) | 12 (52.2) | 0.852 |
| Hypertensive [n(%)] | 13 (28.3) | 16 (34.8) | 0.071 | 3 (13.0) | 7 (30.4) | 0.041 |
| Sport [n(%)] | 13 (28.3) | 12 (26.1) | 0.886 | 8 (34.8) | 9 (39.1) | 0.455 |
| BMI ($kg/m^2$) | 24.9 (3.9) | 25.4 (4.1) | 0.439 | 24.0 (4.3) | 24.8 (3.2) | 0.274 |
| SBP (mmHg) | 140.2 (22.4) | 144.0 (23.3) | 0.022 | 123.3 (15.0) | 125.9 (14.1) | 0.046 |
| DBP (mmHg) | 85.3 (12.7) | 86.2 (15.7) | 0.110 | 79.6 (10.2) | 78.1 (12.2) | 0.442 |
| FPG (mmol/L) | 4.6 (1.0) | 4.8 (1.1) | 0.214 | 4.1 (0.6) | 4.3 (0.6) | 0.321 |
| 2-hPG (mmol/L) | 6.2 (1.7) | 6.3 (2.6) | 0.130 | 5.1 (1.2) | 5.3 (1.3) | 0.256 |
| eGFR (ml/min/1.73 $m^2$) | 110.4 (32.5) | 92.5 (30.6) | <0.001 | 127.7 (34.8) | 116.9 (30.5) | 0.019 |

Continuous variables are presented as the means (SD). Generalized linear mixed models and $\chi^2$ test were used to probe for differences in continuous and dichotomous variables. eGFR, estimated glomerular filtration rate. BMI, Body mass index. FPG, Fast plasma glucose. 2-hPG, 2-hour postprandial glucose. SBP, Systolic blood pressure. DBP, Diastolic blood pressure.

**Supplementary Table 2. Basic characteristics of the studied subjects for targeted bisulfite sequencing analysis.**

| Characteristics | Studied subjects for targeted bisulfite sequencing analysis | | | | | |
|---|---|---|---|---|---|---|
| | F1 parent generation | | | F2 offspring generation | | |
| | Non-famine (n=196) | Famine (n=194) | *p* | Non-famine (n=127) | Famine (n=83) | *p* |
| Age (years) | 52.1 (4.8) | 52.3 (2.7) | <0.01 | 25.8 (4.7) | 26.4 (3.7) | 0.221 |
| Men [n(%)] | 102 (52.0) | 105 (54.1) | 0.554 | 59 (55.1) | 59 (57.3) | 0.754 |
| Energy( kcal/d) | 2424 (1062) | 2477 (1003) | 0.325 | 2578 (883) | 2524 (863) | 0.546 |
| Over high school level [n(%)] | 28 (14.3) | 34 (17.5) | 0.630 | 16 (14.9) | 21 (20.4) | 0.254 |
| Low working strength [n(%)] | 66 (33.7) | 60 (30.9) | 0.362 | 37 (34.6) | 30 (29.1) | 0.523 |
| Smoker [n(%)] | 50 (25.5) | 52 (26.8 ) | 0.884 | 22 (20.6) | 24 (23.3) | 0.215 |
| Drinker [n(%)] | 58 (29.6) | 60 (30.9) | 0.361 | 32 (29.9) | 32 (31.1) | 0.225 |
| Hypertensive [n(%)] | 16 (8.2) | 20 (10.3) | 0.421 | 9 (8.4) | 9 (8.7) | 0.668 |
| Sport [n(%)] | 81 (41.3) | 92 (47.4) | 0.026 | 24 (22.4) | 24 (23.3) | 0.658 |
| BMI (kg/m$^2$) | 24.6 (3.5) | 24.9 (3.9) | 0.332 | 24.1 (3.2) | 24.5 (4.1) | 0.556 |
| FPG (mmol/L) | 4.5 (1.2) | 4.6 (1.1) | 0.331 | 4.2 (0.3) | 4.3 (0.4) | 0.089 |
| 2-hPG (mmol/L) | 6.1 (1.7) | 6.4 (2.8) | 0.044 | 5.2 (1.1) | 5.5 (1.2) | 0.032 |
| SBP (mmHg) | 139.1 (25.6) | 142.7 (24.3) | 0.112 | 125.6 (18.4) | 128.6 (16.5) | 0.245 |
| DBP (mmHg) | 81.3 (15.2) | 83.1 (15.4) | 0.448 | 78.9 (12.3) | 78.8 (14.8) | 0.221 |
| eGFR (ml/min/1.73 m$^2$) | 112.1 (23.5) | 95.6 (22.7) | <0.001 | 124.8 (35.2) | 112.1 (26.6) | 0.029 |

Continuous variables are presented as the means (SD). Generalized linear mixed models and $\chi^2$ test were used to probe for differences in continuous and dichotomous variables. eGFR, estimated glomerular filtration rate. BMI, Body mass index. FPG, Fast plasma glucose. 2-hPG, 2-hour postprandial glucose. SBP, Systolic blood pressure. DBP, Diastolic blood pressure.

**Supplementary Table 3. Target regions for bisulfate sequencing at validation stage.**

| CPG | Gene | Chr | Start | End | Strand | Length | PrimerF | PrimerR |
|---|---|---|---|---|---|---|---|---|
| cg13528513 | AGTR1 | 3 | 148418596 | 148418335 | - | 262 | GTGTTGGAAAGTTG ATTTGTTAGTTT | ATTTACCTAACCCTTTT TCTAATCAATC |
| cg20906621 | AGTR1 | 3 | 148416471 | 148416214 | - | 258 | TTTGGTTAAGGTTG GAGTGATGTT | ACAATTCAATAAACTT TCTTCCTAAAAC |
| cg17160506 | PRKCA | 17 | 64649456 | 64649609 | + | 154 | AGTTTTAGTTATTTA GGAAGTTGAGTGG | AACCTCCTAAATAACT AAAACTACAAATACAC |

**Supplementary Table 4. 19 differentially methylated positions (DMPs) showed concordant alterations in both generations.**

| CPG | Gene Symbol | Chr | Start | End | Relation to gene region | F1 generation | | F2 generation | | Hyper- or Hypo-methylated |
|---|---|---|---|---|---|---|---|---|---|---|
| | | | | | | Δβ | Adjusted *P*-value | Δβ | Adjusted *P*-value | |
| cg03671207 | CCR6 | 6 | 167544220 | 167545175 | 5'UTR | 0.057 | 0.020 | 0.066 | 0.031 | Hyper |
| cg04736673 | CCDC6 | 10 | 61646683 | 61647185 | Body | -0.080 | 0.045 | -0.134 | 0.006 | hypo |
| cg04810224 | PRKG1 | 10 | 53620080 | 53620355 | Body | 0.056 | 0.000 | 0.071 | 0.011 | Hyper |
| cg06332058 | | 9 | 16103640 | 16104095 | | -0.063 | 0.011 | -0.069 | 0.028 | hypo |
| cg09762515 | CUX1 | 7 | 101556445 | 101557055 | Body | 0.051 | 0.014 | 0.074 | 0.008 | Hyper |
| cg13528513 | AGTR1 | 3 | 148418205 | 148418530 | 5'UTR | -0.052 | 0.019 | -0.066 | 0.012 | hypo |
| cg13598358 | PPP1CC | 12 | 111176400 | 111176895 | Body | 0.058 | 0.031 | 0.064 | 0.009 | Hyper |
| cg14032856 | PLA2R1 | 2 | 160839385 | 160839590 | Body | -0.053 | 0.019 | -0.067 | 0.013 | hypo |
| cg15824303 | | | | | | 0.054 | 0.006 | 0.082 | 0.020 | Hyper |
| cg16376748 | FDXR | 17 | 72870025 | 72870515 | TSS1500 | 0.053 | 0.016 | 0.065 | 0.025 | Hyper |
| cg17160506 | PRKCA | 17 | 64649040 | 64649570 | Body | -0.053 | 0.008 | -0.091 | 0.001 | hypo |
| cg17198994 | FGF12 | 3 | 192128785 | 192129475 | Body | 0.063 | 0.008 | 0.074 | 0.013 | Hyper |
| cg18416503 | ITPK1 | 14 | 93475840 | 93476235 | Body | -0.064 | 0.016 | -0.086 | 0.021 | hypo |
| cg20906621 | AGTR1 | 3 | 148416100 | 148416355 | 5'UTR | -0.056 | 0.003 | -0.069 | 0.002 | hypo |
| cg21532512 | ELMO1 | 7 | 37114000 | 37114355 | Body | -0.056 | 0.016 | -0.078 | 0.040 | hypo |
| cg23008606 | ADRA1A | 8 | 26722485 | 26722995 | TSS200 | -0.059 | 0.009 | -0.064 | 0.035 | hypo |
| cg24160354 | PPARGC1A | 4 | 23865508 | 23867708 | Body | 0.061 | 0.023 | 0.105 | 0.023 | Hyper |
| cg26700447 | CCKBR | | | | Body | 0.059 | 0.011 | 0.083 | 0.015 | Hyper |
| cg27631597 | IQGAP2 | 5 | 75903265 | 75903530 | Body | -0.059 | 0.010 | -0.087 | 0.018 | hypo |

19 F1 DMSs showed concordant alterations in F2 cohort were identified by using Illumina Infinium Human Methylation 850K chip. The hypomethylated and hypermethylated DMPs are highlighted with -Δβ and +Δβ, respectively. Δβ refers to the difference in mean methylation β (β-values) between famine group and non-famine group. Chr, chromosome.

**Supplementary Table 5. Target bisulfate sequencing for cg13528513, cg20906621 and cg17160506 in both generations.**

| Product | Chr | Position | Genome Position | Gene Symbol | PrimerF | PrimerR | F1 generation | | F2 generation | |
|---|---|---|---|---|---|---|---|---|---|---|
| | | | | | | | Methylation (Δβ) | P-value | Methylation (Δβ) | P-value |
| cg13528513 | 3 | 136 | 148418461 | AGTR1 | GTGTTGGAAAGTTGATTTGTTAGTTT | ATTTACCTAACCCTTTTTCTAATCAATC | -0.059 | 0.024 | -0.057 | 0.020 |
| cg20906621 | 3 | 42 | 148416430 | AGTR1 | TTTGGTTAAGGTTGGAGTGATGTT | ACAATTCAATAAACTTTCTTCCTAAAAC | -0.078 | 0.015 | -0.062 | 0.033 |
| cg17160506 | 17 | 85 | 64649540 | PRKCA | AGTTTTAGTTATTTAGGAAGTTGAGTGG | AACCTCCTAAATAACTAAAACTACAAATACAC | -0.071 | 0.022 | -0.066 | 0.019 |

The hypomethylated and hypermethylated DMPs are highlighted with -Δβ and +Δβ, respectively. Δβ refers to the difference in mean methylation β (β-values) between famine group and non-famine group. Chr, chromosome. PrimerF, forward primer sequence. PrimerR, reverse primer sequence.
